# Comparison of the risk of obesity in the FTO rs9939609 genotype in a multiethnic group in Asia systematic review and meta-analysis

**DOI:** 10.3389/fmed.2025.1522318

**Published:** 2025-02-06

**Authors:** Donna Pratiwi, Miko Sidartha, Elvan Wiyarta, I Wayan Agustinus Harimawan, Ni Made Dwi Asti Lestari, Bonglee Kim, Nurpudji Astuti Taslim, Trina Ekawati Tallei, Fahrul Nurkolis, Rony Abdi Syahputra

**Affiliations:** ^1^Department of Clinical Nutrition, Ternate Regional General Hospital, Ternate, Indonesia; ^2^Department of Internal Medicine, Bhayangkara Police Hospital Ternate, Ternate, Indonesia; ^3^Critical Care Department, University of Indonesia Hospital, Depok, Indonesia; ^4^Service Department, Risetku, South Jakarta, Jakarta, Indonesia; ^5^Department of Clinical Nutrition, Prof.dr.I.G.N.G Ngoerah General Hospital, Bali, Indonesia; ^6^Department of Clinical Nutrition, Bali Mandara General Hospital, Bali, Indonesia; ^7^Department of Pathology, College of Korean Medicine, Kyung Hee Universitygu, Seoul, Republic of Korea; ^8^Department of Clinical Nutrition, Faculty of Medicine, Hasanuddin University, Makassar, Indonesia; ^9^Department of Biology, Faculty of Mathematics and Natural Sciences, Sam Ratulangi University, Manado, Indonesia; ^10^Department of Biological Sciences, State Islamic University of Sunan Kalijaga (UIN Sunan Kalijaga), Yogyakarta, Indonesia; ^11^Department of Pharmacology, Faculty of Pharmacy, Universitas Sumatera Utara, Medan, Indonesia

**Keywords:** obesity risk, FTO gene rs9939609, Asia, multiethnic groups, AA genotype

## Abstract

**Aim:**

This study aims to examine the comparative risk of obesity in the FTO rs9939609 genotype in multiethnic groups in Asia, considering that obesity has become a global disease.

**Data synthesis:**

Data searches were carried out in several electronic databases: PubMed, Scopus, Google Scholar, ScienceDirect, ClinicalTrials.gov and NCBI. The search involved a combination of keywords related to genetics and obesity risk. Pooled Odds Ratio (POR) with 95% CI was calculated based on the pooled data. Review Manager (RevMan) 5.4.1 were used to analyze the data.

**Results:**

From 18 studies, the results of the dominant genetic model AA vs. TT showed POR 95%Cl = 1.95 (1.36–2.80); *p* < 0.00001, in AA vs. TA genetic recessive model, POR 95%Cl =1.31 (1.07–1.60); *p* = 0.002, then the final model of TA vs. TT codominance genetic model obtained POR 95%Cl = 1.52 (1.04–2.23); *p* < 0.00001. The overall risk of bias was low.

**Conclusion:**

From this research, it was found that there was a comparison of the genotype that had a higher risk of obesity, namely the AA genotype in multiethnic groups in Asia.

**Systematic review registration:**

https://www.crd.york.ac.uk/prospero/display_record.php?RecordID=546434.

## 1 Introduction

Obesity causes physiological and hormonal changes in the body that can trigger many diseases, including diabetes and cardiovascular disease. The medical procedures that have been carried out to treat obesity are when patients arrive already experiencing obesity or even other complications related to obesity. The incidence of obesity in Southeast Asia and South Asia is expected to double from 2010 to 2030. Not only adults, but children are also predicted to increase along with the increase in adults. This increase in incidence could affect around 45 million children in Southeast and South Asia who are over 5 years old (1).

Many factors underlie the occurrence of obesity in human individuals, ranging from genetics, environment and lifestyle. Obesity in humans is 40–70% influenced by genetic factors (2). Genetic factors that influence obesity are the fat mass and obesity (FTO) genes. Someone who has the FTO rs9939609 gene variation prefers foods that have a higher density and can cause weight gain (3). FTO genes have been identified as major genetic risks at obesity loci. The location of the FTO gene itself is at 16q12.2 and has 9 intron and 8 exons. The most strongly associated with obesity and increased body mass index (BMI) is the first intron of FTO (4).

Based on previous research, there is a relationship between polymorphisms of the FTO gene and obesity not only in Asian ethnic group but also in Caucasian and Hispanic (5). They compared the risk of obesity between genotypes only in Asian and Western adult’s populations, whereas we compared all ages from various Asian population. Apart from that, a study was also found which stated that in one tribe in Indonesia, genotype had no effect on the risk of obesity (6). Main reason for this research was carried out because considering the world’s population, especially in Asia we generated the next genotype hypothesis from the FTO rs9939609 gene variation which has a higher risk of obesity, so that it can be used as a preventive measure, early management of lifestyle and diet related to the risk of obesity from variants that contribute to the risk of obesity.

## 2 Materials and methods

### 2.1 Search strategy

The guidelines used by this research are the guidelines described in the Preferred Reporting Items for Systematic Reviews and Meta-Analyses (PRISMA) (7). On June 27, 2024, we have registered this study with PROSPERO (Registered number CRD42024546434). Several databases are used to search for data such as: Scopus, PubMed, Google Scholar, ClinicalTrials.gov, ScienceDirect and NCBI from 2008 up to March 2024. The search using some keywords related to genetics and obesity risk. To identify missed articles, we also searched relevant references, systematic reviews and meta-analyses. Related studies published in PubMed and Google Scholar in English were also included. This research involved populations from various Asian countries such as Indonesia, the Philippines, Japan, China, India, Iraq, Pakistan and Kuwait.

### 2.2 Inclusion and exclusion criteria

Researchers carry out the data search process through electronic devices. Then, an initial screening of the research data was carried out. After that, filtering was carried out to examine the article text thoroughly based on exclusion and inclusion criteria. All articles that meet the inclusion and exclusion criteria will be included in our systematic review and meta-analysis. Researchers use title and abstract as criteria for selecting articles. If inconsistencies occur, each researcher rechecks them.

The researchers used inclusion criteria in Indonesian and English using human subjects and emphasized cross-sectional and case–control studies on the FTO rs9939609 gene variant including the risk of obesity in children and adults in various Asian countries. Exclusion criteria were if it was an animal study, was a case report, did not include gene-diet interactions, the results were not a risk of obesity.

### 2.3 Data extraction

Researchers independently take the necessary information, namely the first are variables related to the main author of the research, publication time, research method, sample size and the existence of a control group. The second evaluates genes, methods for assessing obesity risk, and primary outcomes.

Article data was tabulated with the contents of the main author’s name, year of publication, country of origin, research design, size of sample and genotype. In this research, various ethnic groups in Asia are grouped with Southeast Asia, South Asia, West Asia and East Asia. By using a genetic model based on genotype, a comparison of the relationships can be seen. We divide the genetic model into three models, namely the dominant (AA vs. TT), the recessive (AA vs. TA), and the codominant (TA vs. TT).

### 2.4 Quality assessment

**Figure 1:**
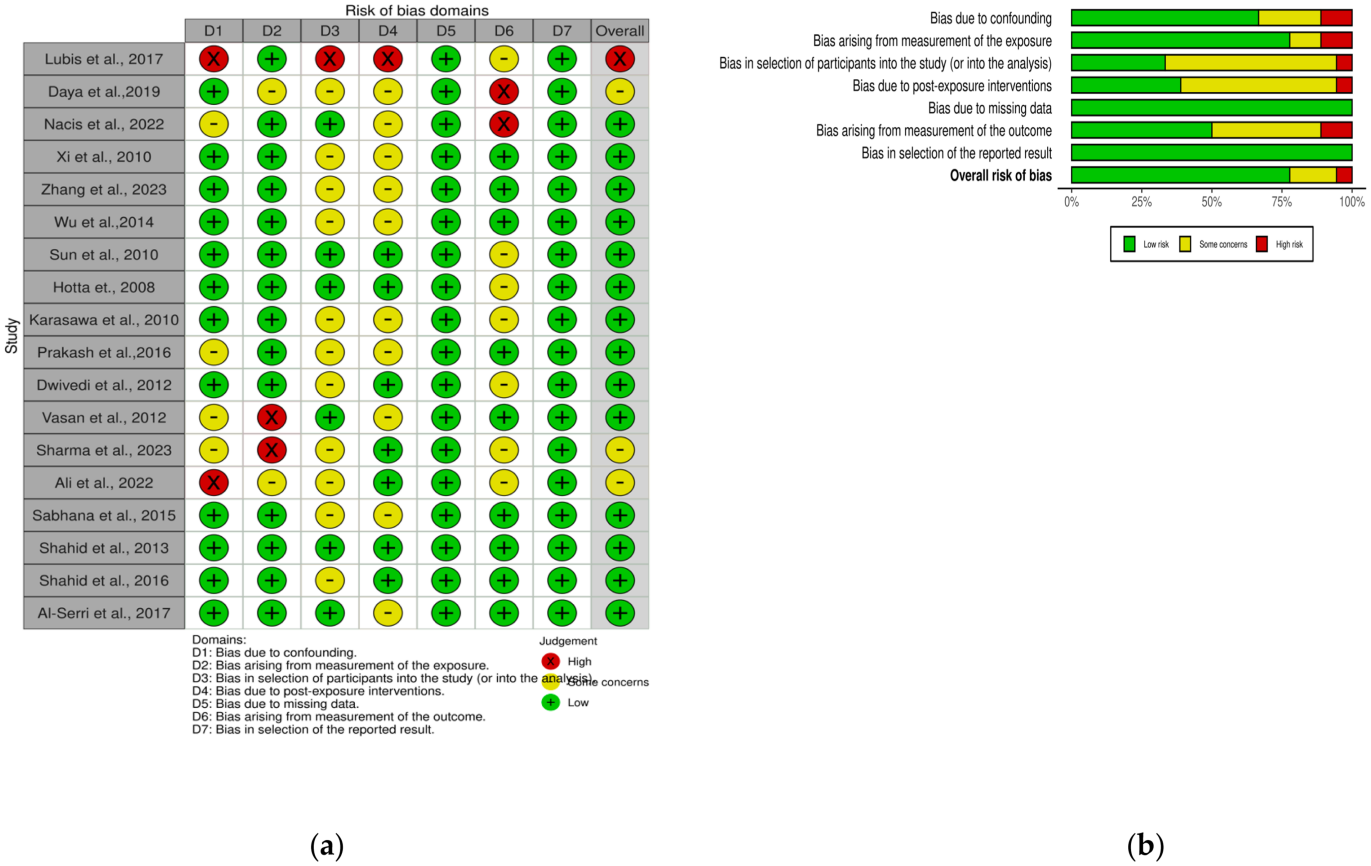
Risk of bias of the entire study **(A)** ROBINS-E Traffic-light plot; **(B)** ROBINS-E Summary plot.

We used the Risk of Bias in Nonrandomized Exposure Studies method or the so-called ROBINS-E tool to assess bias in each included study. With ROBINS-E we assess studies by looking at things like confounding factors, exposure measurements, participants in the study, post-exposure interventions, missing data, outcome measures and final results. Reviewers will examine each paper independently, then award points for each domain (Figure 1). If there are differences of opinion on domain points, the reviewers will discuss with each other. The risk of bias in each study will be classified into low, some concern, high and very high according to the criteria of ROBINS-E. This thorough evaluation will help ensure the accuracy of the conclusions synthesized in the observations.

### 2.5 Eligibility criteria

The population included in this analysis is children aged over 2 years and adults, at risk of obesity and variations in the FTO rs9939609 genotype without looking at food intake or eating habits without referring to the risk of obesity. This study explores which genotypes of the FTO rs9939609 variant have a higher risk of obesity than other genotypes in various Asian countries. Pregnant women, incomplete articles, inappropriate and relevant data were included as exclusion criteria.

### 2.6 Statistical analysis

In the meta-analysis stage, we used Review Manager (RevMan) 5.4.1 (8). To explore the genotype of the FTO rs9939609 variant that has a higher risk of obesity, we used the Pooled Odd Ratio (POR) and the corresponding to the 95% Confidence Interval (CI).

To analyze the heterogeneity of all included studies, we used Subgroup analysis to explore the origins of heterogeneity. Sensitivity analysis carried out to evaluate robustness by eliminated studies that has high risk of bias, we used funnel plots to test for potential publication bias. Asymmetric data distribution indicates publication bias.

In RevMan 5.4.1 we want to see how strong the relationship between genotypes is and obesity risk, so we calculate the POR and 95%CI. An I2 value greater than 50% is a form of study variability so we used a random effects model for variable outcome also a fixed effects model for consistent outcome. These results are illustrated using forest plots and funnel plots which were conducted to assess publication bias.

## 3 Results

**Figure 2:**
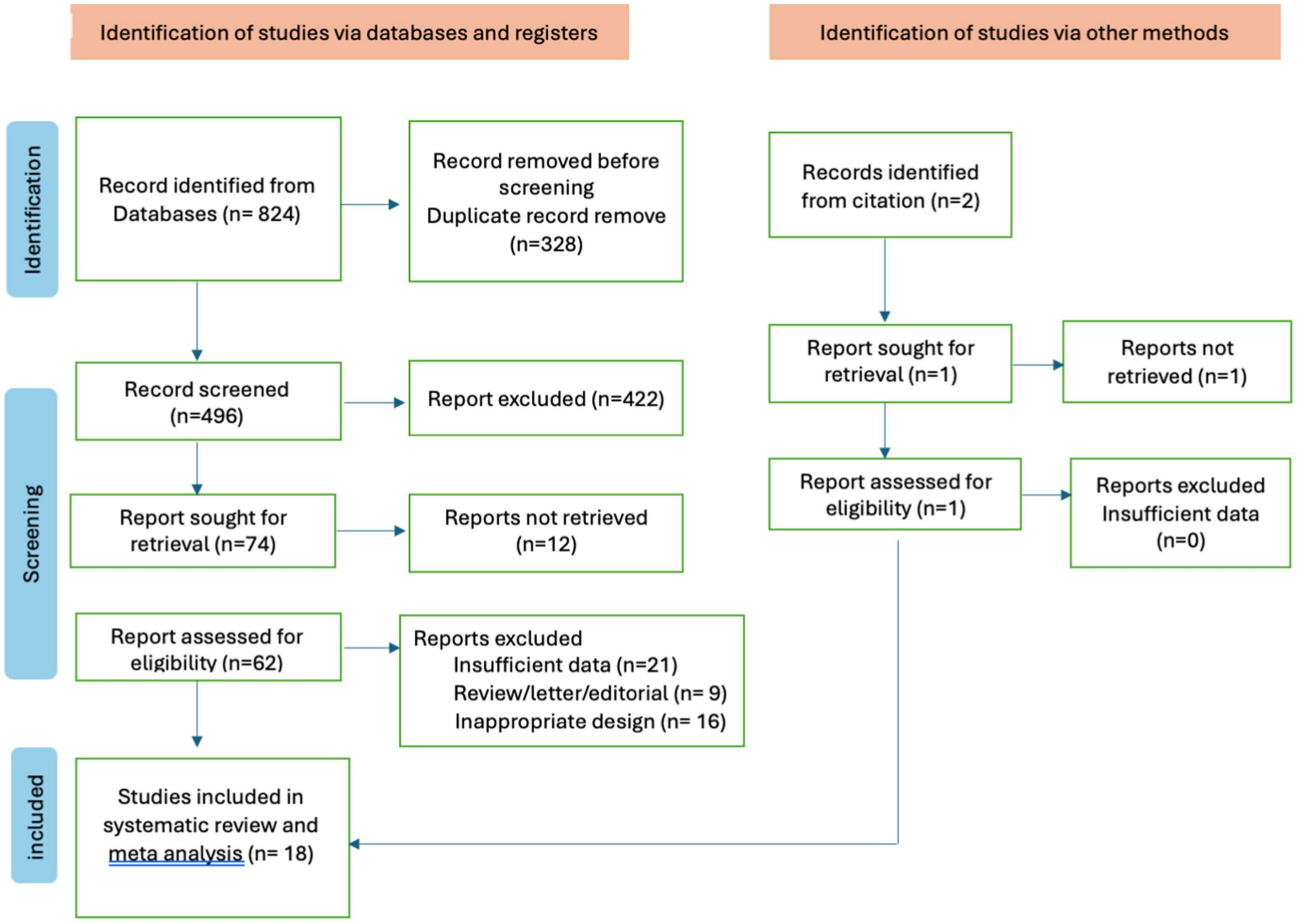
Stages of study selection flowchart for systematic review and meta-analysis.

**Figure 3:**
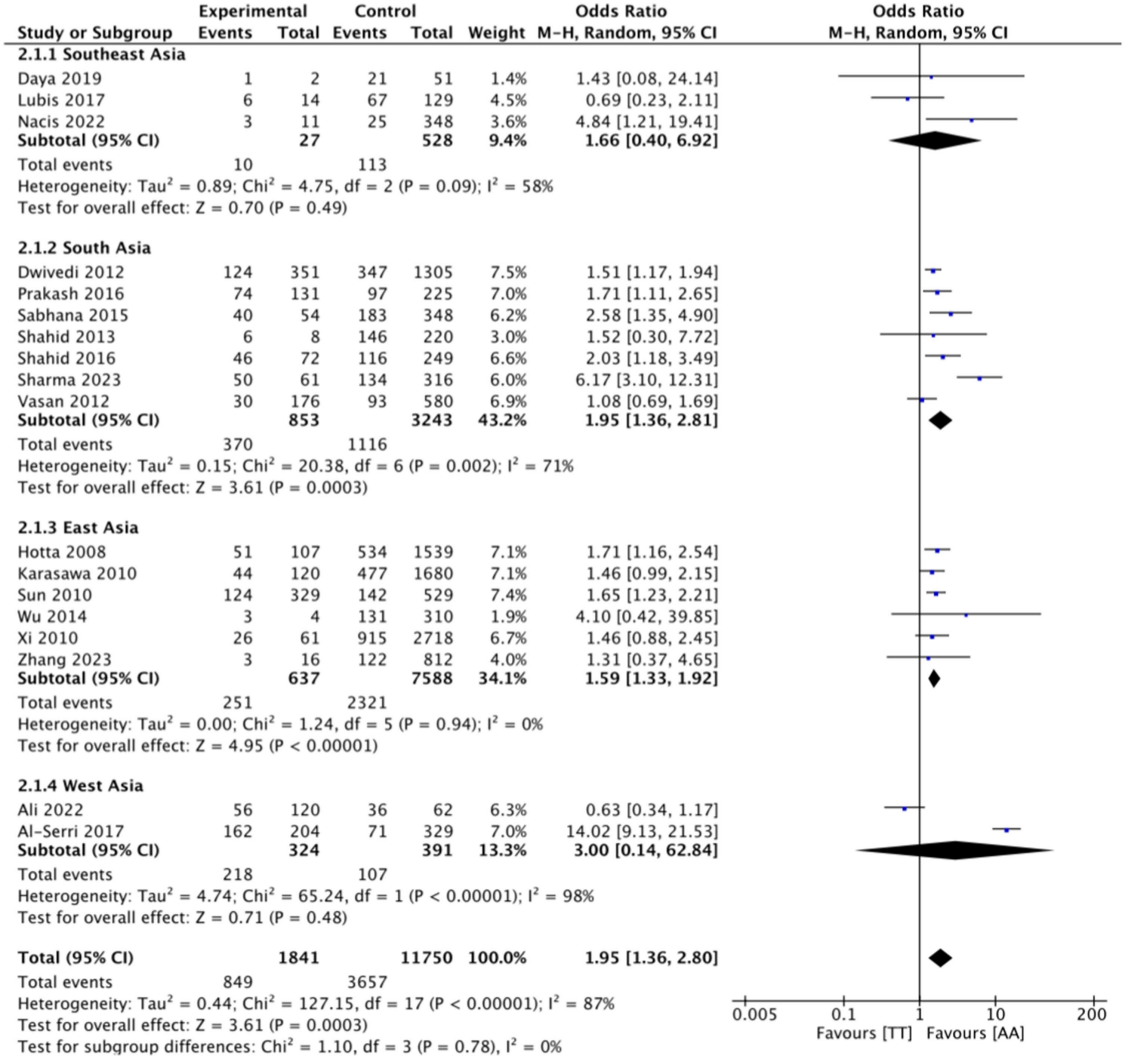
FTO rs9939609 genotype and obesity risk of multiethnic in Asian countries AA vs. TT dominant genetic model.

**Figure 4:**
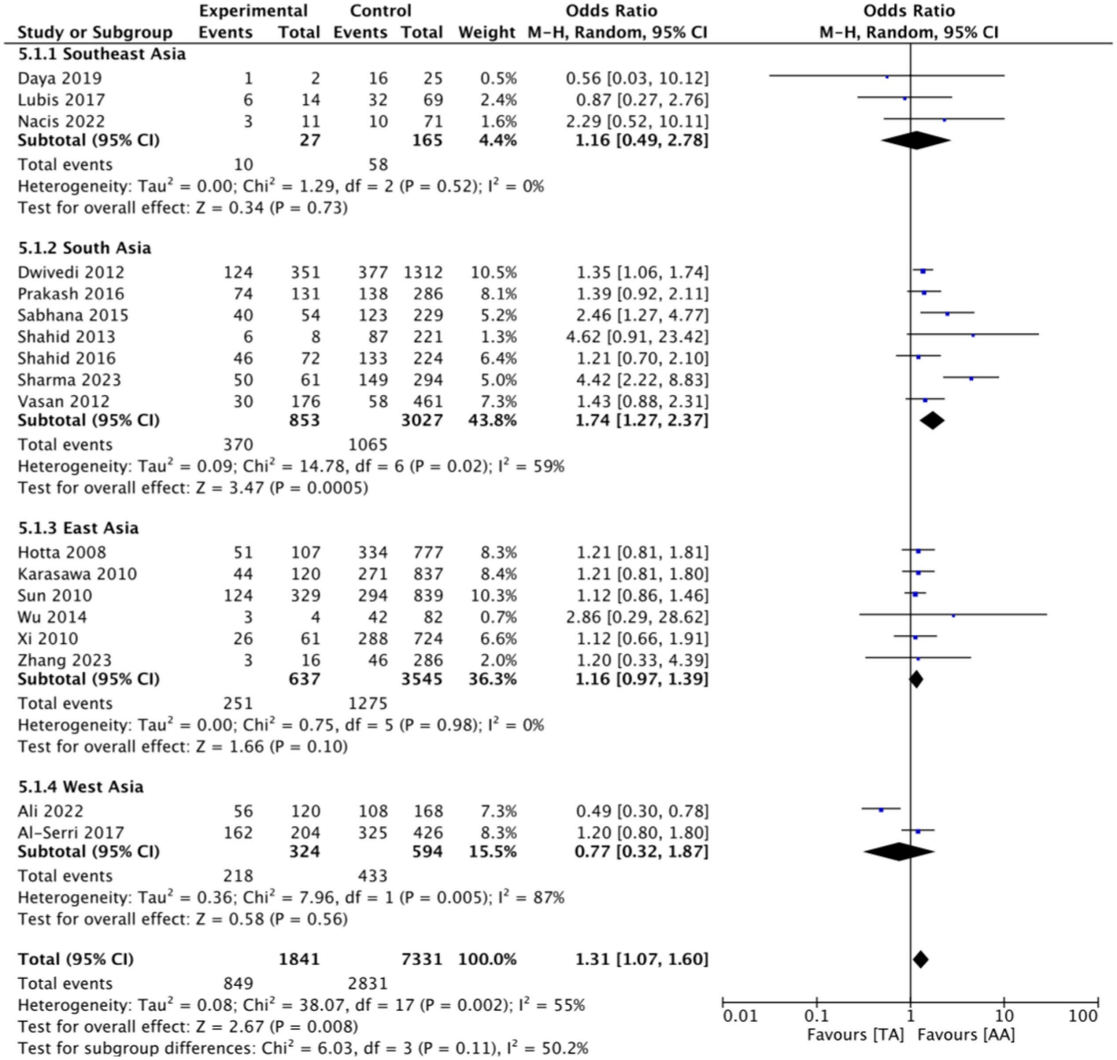
FTO rs9939609 genotype and obesity risk of multiethnic in Asian countries AA vs. TA recessive genetic model.

**Figure 5:**
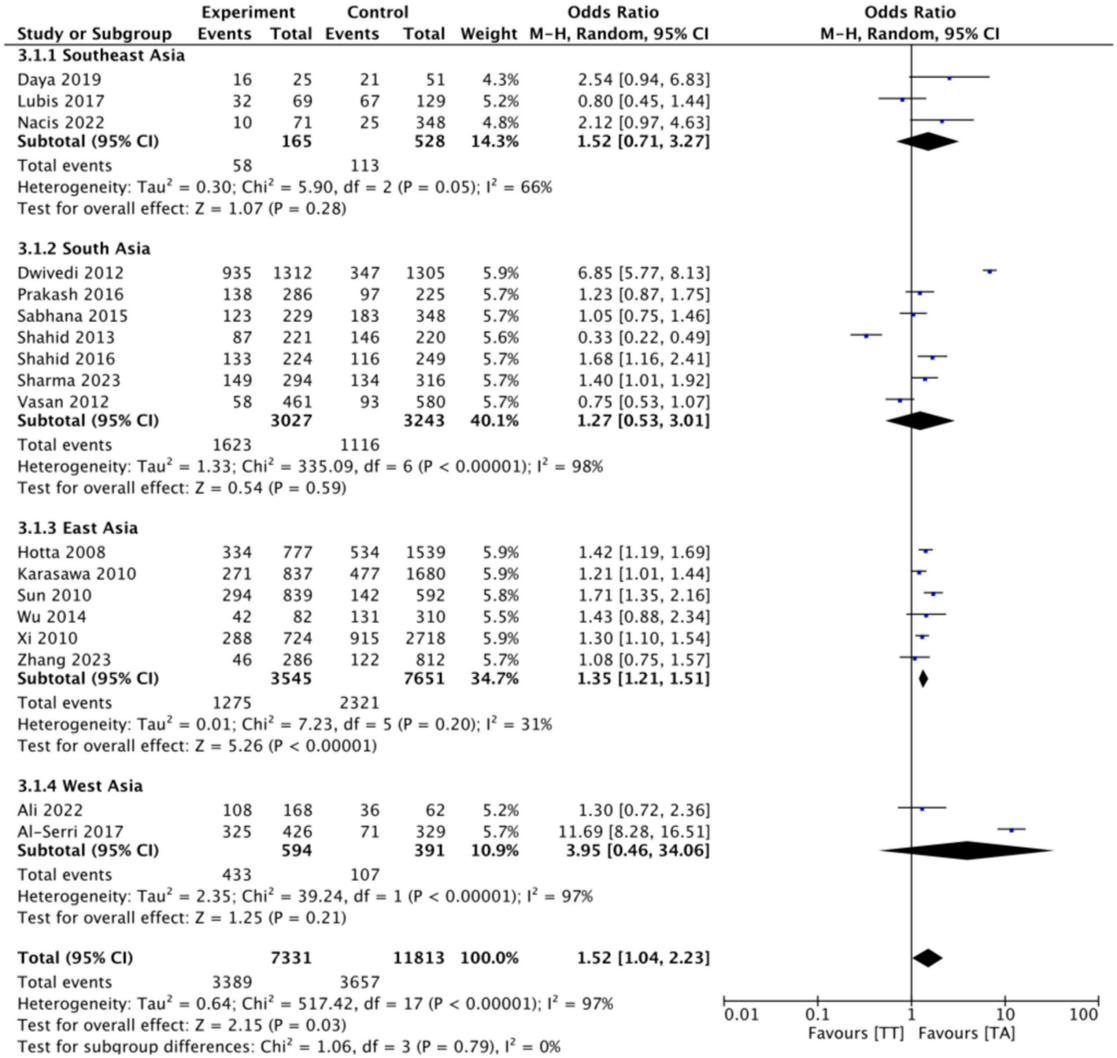
FTO rs9939609 genotype and risk of obesity in multiethnic in Asian countries TA vs. TT codominant genetic model.

This research consists of 18 studies that looked at the risk of obesity due to genetics in the FTO rs9939609 gene variant in various ethnicities in Asia (Table 1). The total sample size included in this study was 20,985 subjects. The study investigated how the polymorphism of FTO rs9939609 gene is related to the obesity risk in children and adults in Asian countries. From all the records that have been searched, researchers sort out which data can be included in the research, including following the inclusion and exclusion criteria. The flowchart of the entire selection process can be seen in Figure 2 and PRISMA checklist can be seen in Supplementary material S1. The results of the AA vs. TT dominant genetic model POR 95%Cl = 1.95 (1.36–2.80); *p* < 0.00001, in AA vs. TA genetic recessive model, POR 95%Cl =1.31 (1.07–1.60); *p* = 0.002, then the final model of TA vs. TT codominance genetic model obtained POR 95%Cl = 1.52 (1.04–2.23); *p* < 0.00001 (Figures 3–5). The results of the risk of bias assessment, we found that there were 5 studies that had a high risk of bias and others had low to moderate risk of bias, which was assessed from 6 domains (Figure 1).

**Table 1:** Studies that have passed the selection and been included in the systematic review of FTO rs9939609 genotype variants and risk of obesity in multiethnic Asians.

Author	Country	Study design	Number of samples		Genotypes					
			Obese	Non-Obese	AA		AT		TT	
					O	NO	O	NO	O	NO
					N %	N %	N %	N %	N %	N %
Lubis et al. (6)	Indonesia	case–control	105	107	6 5.7%	8 7.48%	32 30.48%	37 34.58%	67 63.81%	62 57.94%
Daya et al. (14)	Indonesia	case–control	38	40	1 2.6%	1 2.5%	16 42.11%	9 22.5%	21 55.26%	30 75%
Nacis et al. (20)	Philippine	cross- sectional	38	392	3 8%	8 4%	10 26%	61 30%	25 66%	323 81%
Xi et al. (21)	China	cross-sectional	1,229	2,274	26 2.11%	35 1.54%	288 23.43%	436 19.17%	915 40.23%	1803 79.28%
Zhang et al. (21)	China	case control	171	1,065	3 1.75%	13 1.22%	46 26.9%	240 22.54%	122 71.35%	812 76.24%
Wu et al. (22)	China	case control	176	220	3 1.7%	1 0.45%	42 23.86%	40 18.18%	131 74.43%	179 81.36%
Sun et al. (23)	China	case control	560	1,200	124 22.1%	205 17.1%	294 52.9%	545 45.5%	142 25.4%	450 37.5%
Hotta et al. (24)	Japan	case control	919	1,504	51 5.55%	56 3.72%	334 36.34%	443 29.45%	534 58.1%	1,005 66.82%
Karasawa et al. (25)	Japan	case control	794	1845	44 5.54%	76 4.12%	271 34.13%	566 30.67%	477 60.07%	1,203 65.2%
Prakash et al. (26)	India	case control	309	333	74 23.94%	57 17.11%	138 44.66%	148 44.4%	97 31.39%	128 38.43%
Dwivedi et al. (27)	India	cross sectional	848	2,147	124 14.62%	227 10.57%	377 44.45%	935 43.54%	347 40.91%	985 45.87%
Vasan et al. (11)	India	cross sectional	181	676	30 16.57%	146 14.09%	58 8.58%	403 38.89%	93 51.38%	487 47%
Sharma et al. (12)	India	case control	333	338	50 15%	11 3.25%	149 44.7%	145 42.89%	134 40.24%	182 53.8%
Ali et al. (13)	Iraq	case control	200	150	56 28%	64 42.7%	108 54%	60 40%	36 18%	26 17.3%
Sabhana et al. (28)	Pakistan	case control	346	285	40 (11.5%)	14 (5%)	123 (35.7%)	106 (37.1%)	183 (52.9%)	165 (57.9%)
Shahid et al. (29)	Pakistan	case control	239	130	6 (2%)	2 1%	87 36%	34 26%	146 61%	74 72%
Shahid et al. (30)	Pakistan	case control	295	250	46 15.59%	26 10.4%	133 45.08%	91 36.3%	116 39.3%	133 53.2%
Al-Serri et al. (31)	Kuwait	case control	674	214	162 24%	42 20%	325 (48.5%)	101 (47%)	71 (33%)	258 (29%)

### 3.1 Heterogeneity and sensitivity analysis

To analyze potential sources of heterogeneity, we performed further subgroup analysis. All studies were grouped based on ethnicity, Southeast Asia, South Asia, West Asia and East Asia. Based on this, it was found that East Asian countries in the AA VS TT model obtained *p* = 0.94, in the AA VS TA model obtained *p* = 0.98 and TA VS TT obtained *p* = 0.20. Meanwhile, for studies from other countries, results were > 0.01 (Figures 3–5).

To see the robustness of this research, we carried out a sensitivity test. We eliminated studies that could make the results inaccurate. We excluded six studies that had “high risk” results in several domains (9–14), after we excluded the results of AA vs. TT dominant genetic model POR 95%Cl was 2.12 (1.87–2.39), in AA vs. TA genetic recessive model, POR95 %Cl is 1.27(1.12–1.43), then the final model of TA vs. TT codominance genetic model obtained POR95%Cl is 1.44(1.34–1.54) (Supplementary material S2). Overall, these results only make a small difference from the results before elimination, so that the current research can still be said to be robust and fit.

### 3.2 Publication bias

**Figure 6:**
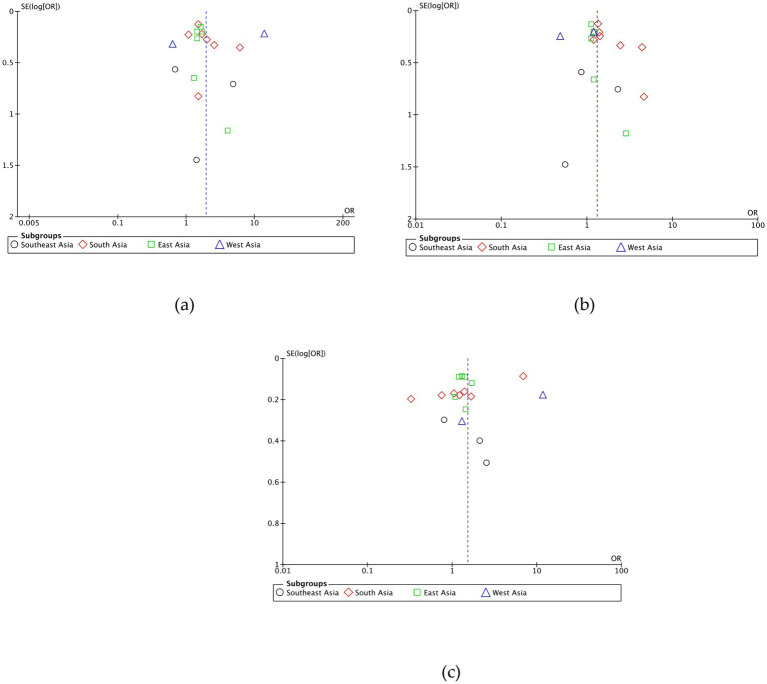
**(A)** Funnel plot AA VS TT. **(B)** Funnel plot AA VS TA. **(C)** Funnel plot TA VS TT.

On the result of the asymmetric funnel plot test, most studies show symmetric results (Figure 6), which means there is no publication bias across the studies.

## 4 Discussion

This research has proven that certain genotypes variant FTO rs9939609 have a different risk of obesity between one genotype and another. The risk of obesity in humans is associated with genetic polymorphisms in the non-coding region of the FTO locus. Even though the numbers are small, the impact is statistically significant. Previous research stated that the FTO variation had a 23% greater risk of obesity (15).

The FTO gene in the human body is on chromosome 16q12.2 which is 41.50 kb long and consists of 8 exons and 9 introns. The most widespread expression of the FTO gene is in the arcuate nucleus of the hypothalamus, where the expression plays a role in measuring energy balance. Apart from that, FTO is also distributed in adipose tissue and skeletal muscle tissue. Because of the distribution in this location, it is possible that the FTO gene has a role in regulating calorie needs through appetite and energy metabolism (16). In the human DNA sequence, single nucleotide polymorphism sites or also called SNPs are the main form of variation and function to regulate gene expression. Changes in gene expression caused by demethylation of nucleic acids from the FTO protein can then increase the risk of obesity and other obesity-related diseases.

The three SNPs in the first intron of the FTO gene that are most found in the Indonesian population are rs9939609, rs1421085 dan rs17817449. In Indonesia, the variant most studied among various ethnicities is the FTO rs9939609 variant with allele A and genotype AA as risk factors for obesity. These minor alleles significantly increase the risk of obesity (17). The FTO variant (rs9939609) is the most closely associated locus GWAS for BMI in 7,861 Koreans including ethnic Malays living in Singapore, this variant is strongly related with obesity (18). Apart from this, this variant is also related with increased calorie intake in adults. Individuals with the AA or TA genotype consume higher calorie foods than the TT genotype in ethnic groups in Southeast Asia, including Indonesia (19). The risk allele A (AA or TA) at rs9939609 may also modulate the preference for high-fat food consumption in adults.

It appears that the TT genotype in the FTO rs9939609 gene variation in Asians has a lower risk of obesity in this study. The AA genotype increases the risk of obesity 1.6 times greater than the TT genotype and 1.57 times greater than the TA. However, this research has limitations, especially due to the lack of data from other ethnicities in Asia. The studies reviewed are mostly case–control studies that have limited sample sizes, public health habits and certain gene variations with obesity are also very important in various populations (5). The development of plans for the prevention and therapy of obesity must be further improved because the association between the FTO rs9939609 gene variation has varying strengths depending on the genotype and population. Adjusting interventions to consider variations that are more influential in obesity risk may result in more successful strategies for preventing and treating obesity. In Asian countries this is especially relevant, where the risk of obesity and certain genotypes can be stronger. The results of this study show that interventions that can target genotypes that have a higher risk of obesity can be a preventive trick that reduces vulnerability to obesity in populations in Asia. Genetic factors can also influence the amount of food consumption, adipogenesis and energy expenditure. It can be underlined that from this research, a personal approach for each individual is needed to prevent and treat obesity. Do not forget to also consider specific genetic variations as genetic factors.

There are several limitations of this study such as some of the results of the included studies are still heterogeneous and there is publication bias in some outcomes. Also, cross-sectional and case–control designs are prone to selection bias and may not establish causality (32). Looking at the risk of bias, most of the results are low to moderate risk of bias, it can be concluded that the strength of this study is good and robust.

In the future, this approach will be able to produce more effective methods to reduce the incidence of obesity and all the health risks associated with obesity, therefore it is important for further research to be carried out including integrate dietary intake, physical activity, and other lifestyle factors into the analysis to provide a more holistic understanding of obesity risk.

## 5 Conclusion

The AA genotype of the rs9939609 genetic variation in various countries has a greater risk of obesity than other genotypes of this variation. This review can be used to assess the risk of obesity based on genotype. Further research is needed to assess obesity risk based on other ethnicities in different parts of the world.

## Data Availability

The raw data supporting the conclusions of this article will be made available by the authors, without undue reservation.
