# Supplementary material for: Comparison of the risk of obesity in the FTO rs9939609 genotype in a multiethnic group in Asia systematic review and meta-analysis
Source: Front Med (Lausanne). 2025 Feb 6;12:1522318. doi: 10.3389/fmed.2025.1522318 (PMC11839718; doi:10.3389/fmed.2025.1522318)
Supplement: Supplementary file 2 [file Data_Sheet_2.docx]

**S2**. Sensitivity Analysis


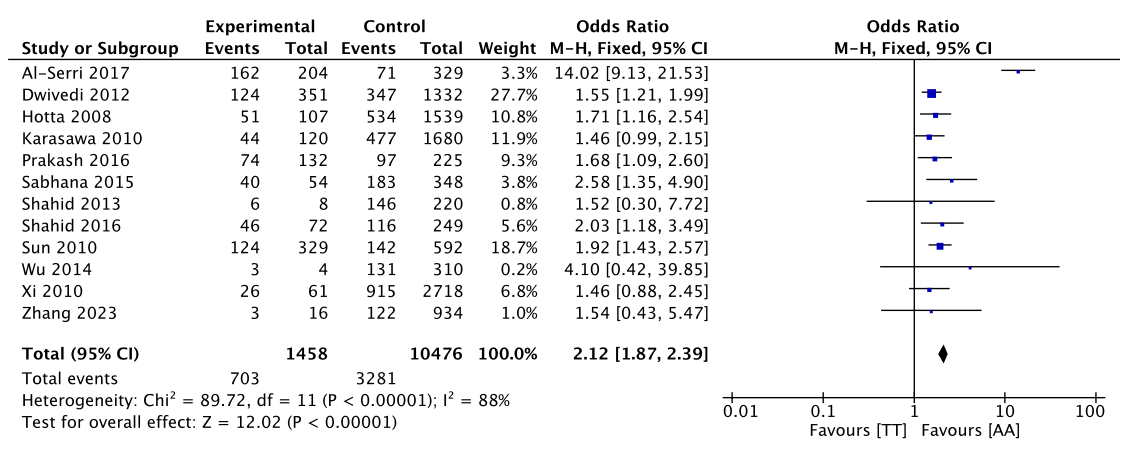


1. Sensitivity analysis FTO rs9939609 genotype and obesity risk of multi ethnic in Asian countries AA vs TT dominant genetic model


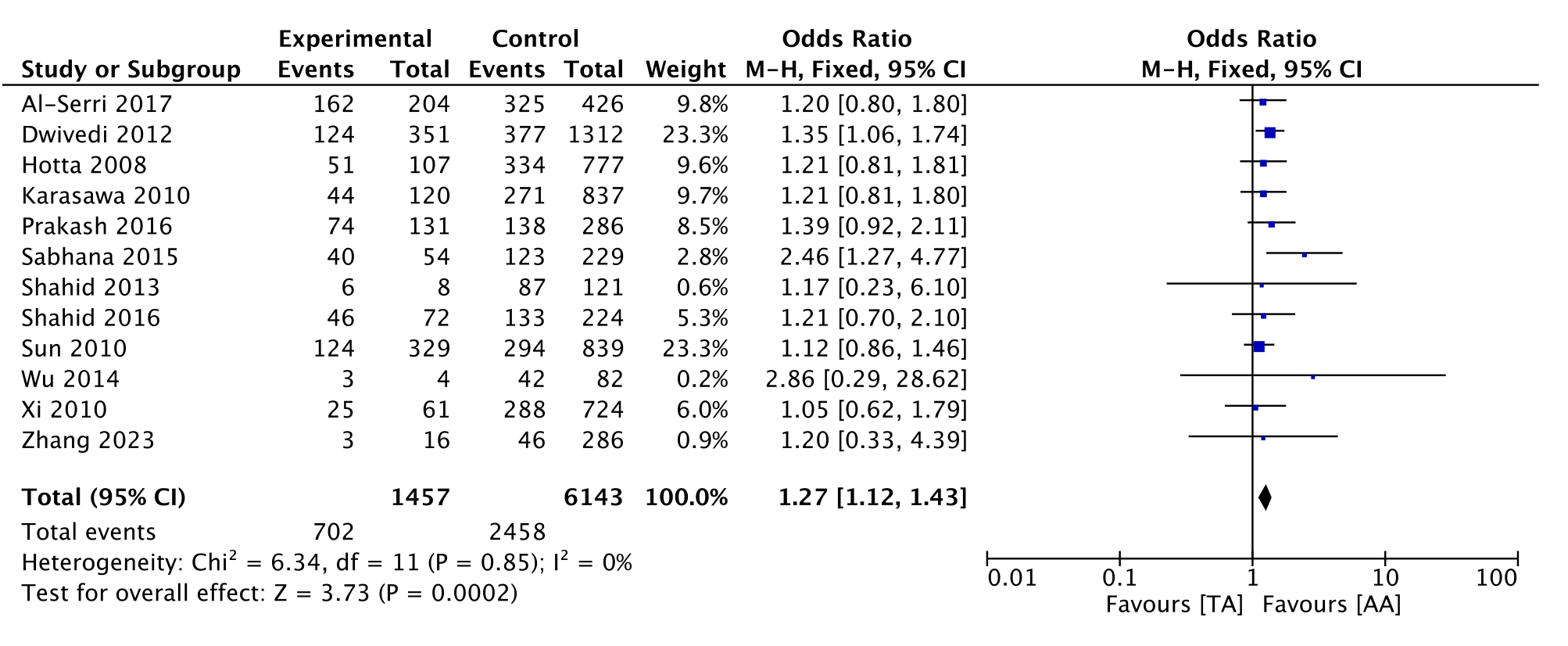


1. Sensitivity analysis FTO rs9939609 genotype and obesity risk of multi ethnic in Asian countries AA vs TA recessive genetic model


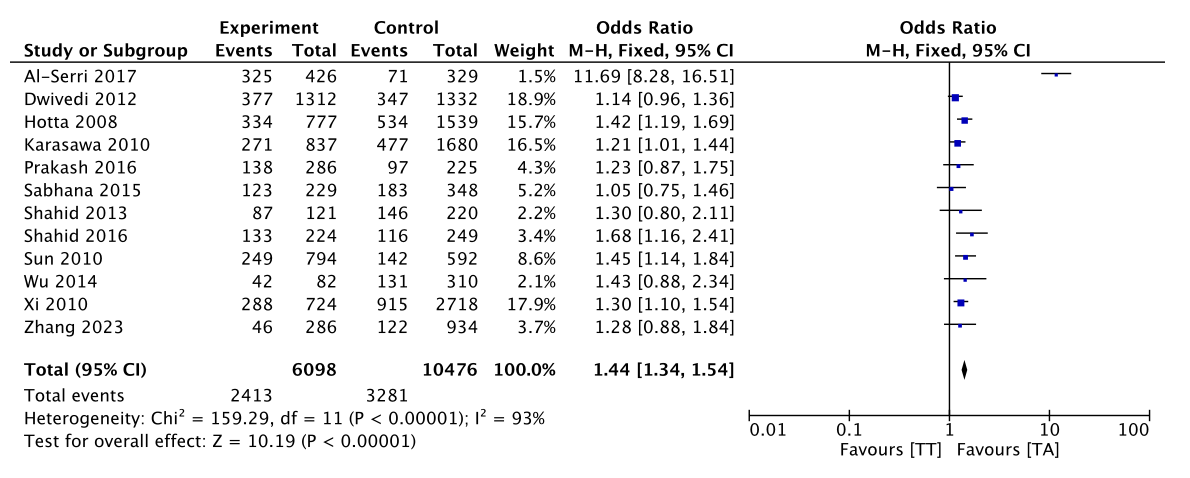


1. Sensitivity analysis FTO rs9939609 genotype and obesity risk of multi ethnic in Asian countries TA vs TT codominant genetic model
